# Using actions to enhance memory: effects of enactment, gestures, and exercise on human memory

**DOI:** 10.3389/fpsyg.2012.00507

**Published:** 2012-11-19

**Authors:** Christopher R. Madan, Anthony Singhal

**Affiliations:** ^1^Department of Psychology, University of AlbertaEdmonton, AB, Canada; ^2^Department of Systems Neuroscience, University Medical Center Hamburg-EppendorfHamburg, Germany; ^3^Centre for Neuroscience, University of AlbertaEdmonton, AB, Canada

Few would doubt the benefits of exercise on one's physical well-being. However, the benefits of exercise on one's mental abilities are not nearly as extolled. More directly, the perspective that our bodies have a significant influence on our minds is still relatively new, though reviews by Rosenbaum (2005) and Madan and Singhal (2012a) suggest that this is beginning to change. This idea is also in line with the embodied approach to cognition (e.g., Clark, 1997; Lakoff and Johnson, 1999; Wilson, 2002; Anderson, 2003; Barsalou, 2008; Fischer and Zwaan, 2008). Briefly, embodied cognition suggests that physical properties of the human body, particularly the perceptual and motor systems, play an important role in cognition—the body influences the mind just as the mind influences the body. This approach is further supported by findings that individual body properties such as handedness can influence how individuals understand abstract concepts (Casasanto, 2009, 2011). One particularly interesting facet of the idea that our body can affect cognition is the influence of actions, gestures, and exercise on memory performance: the hypothesis is that our physical movements, and even the amount that we exercise, can affect our ability to remember. In the current paper we will provide an overview on the disparate research paradigms that support this hypothesis, and their resulting implications.

## Reading, imagining, seeing, and doing

While the majority of studies investigating human memory use words or images as stimuli, Cohen (1981) asked participants to perform actions, to observe an experimenter perform the actions, or simply hear/read the instructions for the actions without it being performed. Participants were subsequently tested for their ability to recall the actions (e.g., “break the tooth-pick”). In later literature, these conditions were termed as self- or subject-performed tasks (SPT), experimenter-performed tasks (EPT), and verbal tasks (VT), respectively. Cohen found that participants were significantly more likely to remember SPTs or EPTs than VTs. Extending this finding, Denis et al. (1991) observed better memory for SPTs than actions that were imagined, testing imagined actions through both visual and motor imagery. Apart from the two studies described here, the work by Cohen and Engelkamp were the beginnings of a new field of research: memory of action events (for reviews, see Engelkamp and Zimmer, 1989; Engelkamp and Cohen, 1991; Zimmer and Cohen, 2001. The primary finding of this field was “the enactment effect”: enhanced memory for SPTs, lending an early source of support to the embodied cognition perspective.

The leading theories proposed to explain the enactment effect were based on two main ideas: (A) Performed actions involve much richer and elaborative representations than mere verbal phrases, and (B) enacted actions engage the motor system whereas other methods of encoding do not. From perspective A, the finding that the enactment enhances memory by serving as an elaborative encoding strategy is also in-line with Craik and Lockhart's (1972) levels-of-processing framework, where information that is processed more deeply/elaboratively is remembered better than information that is only processed relatively shallowly/superficially. However, the nuances by which the enactment effect enhances memory [e.g., lack of a primacy efficacy, some resilience to aging-related attenuations; discussed in Engelkamp and Cohen (1991)] supports the possibility that engaging the motor system during encoding is dissimilar to how information is usually encoded through sensory-based modalities (e.g., visual, auditory; see Engelkamp and Zimmer, 1984; Zimmer et al., 2000).

While the enactment effect itself is noteworthy, it leads to a broader question: under what circumstances can actions enhance or impair memory? Here, a partial answer can be found in a relatively unrelated field: research into gestures.

## Gesture to remember

Gestures are motor actions that often accompany speech, and are intertwined with spoken content (McNeill, 1992; Krauss, 1998; Kelly et al., 2008). Recent findings suggest that gestures may be produced as a type of simulated action that arises when motor activation due to mental imagery processes exceeds a certain threshold (Hostetter and Alibali, 2008; Kelly et al., 2011), in close support of embodied cognition. Additionally, gesturing has been shown to improve problem-solving abilities by decreasing working memory load by conveying the same information through a second, image-based, modality (Morsella and Krauss, 2004; Beilock and Goldin-Meadow, 2010; Cook et al., 2012). Since motor actions enhance memory (enactment effect), it seems reasonable to expect that gesturing may also affect memory encoding and retrieval. Supporting this notion, a number of studies have found that memory is enhanced in participants who observe a speaker who is also gesturing compared to observing a speaker who is not gesturing, or a gesturer who is not speaking (e.g., Thompson, 1995; Kelly et al., 1999). Thus, learning can be enhanced due to gesturing, even when one does not gesture themselves, but simply observes another gesturing. Considering that findings regarding the enactment effect found comparable recall rates with SPTs and EPTs, observing a gesture should be comparable to gesturing yourself.

Taking a more direct approach, Cook et al. (2010) presented participants with a series of short vignettes, after which they were asked to give detailed descriptions. The vignettes were then classified as either eliciting gestures during their description or not. Participants were given surprise free recall tasks after a brief delay, and after a 3-week delay. Recall rates were higher for vignettes associated with gesturing when described at both immediate and delayed tests, offering support for the notion that gestures can enhance learning and memory. In a subsequent experiment, enhanced memory performance was found even when participants were explicitly instructed to either gesture or not, rather than being allowed to spontaneously gesture. Stevanoni and Salmon (2005) found similar results with children, and recent studies have further investigated the influence of gestures on learning and memory (e.g., Straube et al., 2008; Macedonia et al., 2011; So et al., 2012).

Considering that motor actions can enhance memory for specific information, both through enactment and through gesturing, a further question is whether they can also enhance overall memory ability. In other words, can physical exercise enhance an individual's memory capacity?

## Working out your body to expand your mind

While the idea that physical exercise could increase memory recall ability is recent focus of research, it has been shown several decades ago in older adults (Powell, 1974; Diesfeldt and Diesfeldt-Groenendijk, 1977), and has even been shown to lead to enhanced memory abilities as much as one year later (Perrig-Chiello et al., 1998). More recently, daily physical exercise has been shown to reduce the cognitive decline associated with aging as well as reduce the risk of developing Alzheimer's disease (Buchman et al., 2012).

Apart from research on older adults specifically, there is a considerable body of research on the effects of exercise on cognitive performance. Unfortunately, a comprehensive examination at the literature reveals inconsistent findings, with some studies finding an enhancement of cognitive ability due to exercise while others report impairments. A detailed review by Tomporowski (2003) resolves these inconsistencies by accounting for the nature of the physical activity used: intensive exercise to dehydration leads to impairments in cognitive performance, while less intensive, aerobic, exercise leads to enhanced performance, including enhanced memory ability. In addition to behavioral measures of enhanced memory, structural MRI images of the brain before and after week-to-month long exercise protocols have also shown increased hippocampal volume due to the exercise intervention (Pereira et al., 2007; Erickson et al., 2011), extending the results of a number of prior findings in rodents (Uysal et al., 2005; Pereira et al., 2007; Wu et al., 2007; Clark et al., 2011). While it appears clear that exercise has beneficial effects on memory and hippocampal neurogenesis, it should also be noted that the benefits of exercise on cognition are not confined to only memory or the hippocampus, but also extend to a wider range of cognitive processes, particularly executive function and the prefrontal cortex and anterior cingulate cortex (see Hillman et al., 2008, for a review).

Considering the long-term effects of physical exercise on memory ability, as well as the structural changes observed in hippocampal volume, it is worth considering if the opposite is also true: would obesity be correlated with decreases in memory performance? One established indicator of obesity is the body mass index (BMI). Lending some support to this hypothesis, Trakas et al. (2001) found obese individuals to self-report being more forgetful. While this is in-line with our prediction, this result alone is insufficient to evaluate if obesity is affecting memory ability itself, or perhaps just memory confidence, or other-related processes. However, drawing conclusions from two studies that tested this hypothesis directly with batteries of cognitive tasks (Elias et al., 2003; Gunstad et al., 2006), it appears that the answer is “yes”, at least in some cases. In a preliminary analysis, Elias et al. (2003) found no difference in cognitive performance measures for normal weighted and overweight individuals and thus grouped the data for these participants together as “non-obese”. When comparing memory performance for non-obese and obese participants, the obese participants performed worse in some memory tasks, but the effect was only observed in males. Gunstad et al. (2006) classified participants as normal, overweight, or obese and found significant memory impairments in a variety of memory tasks that correlated with BMI (and also not interacting with age). Other studies have used longitudinal analyses, however, results are mixed with some studies finding a relationship (Brubacher et al., 2004) and others finding no correlation (Cournot et al., 2006). Recent research further suggests that hippocampal neurogenesis may also be influenced by diet, insulin levels, and genetic factors (Brubacher et al., 2004; Lindqvist et al., 2006; Nichol et al., 2009; Wallner-Liebmann et al., 2010; Clark et al., 2011; Grillo et al., 2011). While these results are likely not enough to make you think twice about skipping on a run to watch TV, they do suggest that our mind and body may be more closely connected than previously thought—and extend the boundaries commonly applied to embodied cognition.

## Moving forward

Taken together, these unrelated lines of research all lead to one conclusion: our minds and our bodies are more connected than previously thought, and we should not choose between honing *either* our mind or our body. Related research can support this conclusion even further, where movement-related properties (e.g., “affordances”, see Gibson, 1977, 1979) of objects and even words can influence how we process information (e.g., Handy et al., 2003; see Madan and Singhal, 2012a, for a review). In particular, recent findings suggest that the motoric properties of words representing objects, i.e., word manipulability, and how these words are processed can also influence verbal processing Rueschemeyer et al. 2010; also see Just et al., 2010) as well as enhance memory recall (Madan and Singhal, 2012b).

In addition to supporting embodied cognition, the idea that actions enhance memory is also well in-line with the motor chauvinist perspective (Wolpert et al., 2001), where it is hypothesized the brain and, in turn, cognitive function may have evolved to facilitate an organism's ability move within their environment (also see Glenberg, 1997; Gallese and Sinigaglia, 2010; Madan and Singhal, 2012a). If this viewpoint is correct, one would predict that movements should enhance memory function. That is, actions that are executed should be remembered better than those that are read about. Ideas that are communicated in parallel with actions (e.g., gestures) should be remembered better than those that are communicated in the absences of movement. And, general memory ability should be enhanced by physical exercise. Current evidence suggests that all these predictions are valid.

An important idea that has emerged in cognitive science is that the body influences the mind. The embodied cognition approach suggests that motor output is integral to cognition, and the converging evidence of multiple avenues of research further indicate that the role of our body in memory processes may be much more prevalent than previously believed. The extent of this cannot be overstated, and has implications for all memory research. For instance the gesture literature suggests that if a participant were to use gestures while engaged in paired-associate learning, there is the chance that the results could be contaminated with variability due to the gesturing itself. Even more broadly, the majority of studies of memory rely on motor actions to provide behavioral measures of cognition, usually in the form of a button/key press. For example, a widely applied paradigm in cognition, the go/no-go task, requires overt motor responses on some trials and overt inhibition of motor processes on others. However, if the interaction between cognition and motor action is not a one-way process, that is, the action also influences the memory, perhaps amplifying or attenuating the effect size—there is the potential for other inferences to be drawn about the outcome.
